# *BMI1*, *ALDH1A1*, and *CD133* Transcripts Connect Epithelial-Mesenchymal Transition to Cancer Stem Cells in Lung Carcinoma

**DOI:** 10.1155/2016/9714315

**Published:** 2015-12-07

**Authors:** Ana Koren, Matija Rijavec, Izidor Kern, Eva Sodja, Peter Korosec, Tanja Cufer

**Affiliations:** University Clinic Golnik, Golnik 36, SI-4204 Golnik, Slovenia

## Abstract

Epithelial-mesenchymal transition (EMT) is the underlying mechanism of tumor invasion and metastasis. Evidences from lung cancer cellular models show EMT can trigger conversion to a cancer stem cell (CSC) phenotype. In this study, we assessed mRNA expression levels of EMT-inducing transcription factors (*BMI1*, *TWIST1*), CSC (*CD133*, *ALDH1A1*), and epithelial (*EpCAM*) markers in primary tumor and whole blood samples obtained from 57 patients with operable non-small-cell lung cancer (NSCLC) as well as in circulating tumor cells (CTCs) of 13 patients with metastatic disease; then possible associations between marker expressions were evaluated. In primary tumors as well as in whole blood, correlations between *BMI1* and *ALDH1A1* and between *BMI1* and *CD133* mRNA expressions were identified. No correlations between *TWIST1* and CSC markers were observed. *BMI1* mRNA expression in tumors positively correlated with *BMI1* mRNA expression in blood. The immunohistochemical analysis confirmed coexpression of BMI1 and CSC markers in tumors. Gene expression profiling in CTCs revealed upregulated expression of EMT/CSC markers in CTCs. Our results suggest CSCs are present in both, tumor tissue and blood of NSCLC patients, whereas Bmi1 may play an important role in initiation and maintenance of CSCs and might be involved in the blood-borne dissemination of NSCLC.

## 1. Introduction

Lung cancer remains the leading cause of cancer-related mortality in the world and most frequently diagnosed cancer worldwide, with non-small-cell lung cancer (NSCLC) accounting for about 80−85% of all lung cancer cases [1]. Despite substantial diagnostic and therapeutic improvements in the last two decades [2], the overall 5-year survival rate for lung cancer patients is still below 15% [1]. The predominant reason for high mortality rate in lung cancer patients is early tumor spread of lung cancer cells to distant, metastatic sites and primary or acquired resistance of those cells to systemic therapy. Consecutively, more than two-thirds of the patients are diagnosed with locally advanced or metastatic disease and nearly half of the patients who are diagnosed with early stage disease relapse within 5 years after surgical removal of the tumor mass and succumb from widely spread therapy resistant disease [3].

There is a growing body of evidence that cancer stem cells (CSCs) represent rare population of exclusively tumorigenic cells responsible for tumor initiation, progression, metastasis and recurrence [4, 5]. Therefore, a better understanding of the biology of CSCs is providing opportunities for improved cancer detection and therapy in future. Various markers have been proposed to define stem cell populations in distinct solid tumors types [6]. Expression of the cell surface molecule CD133 and high aldehyde dehydrogenase (ALDH) enzymatic activity are well accepted markers for lung CSCs [7]. Both markers independently allow for selection of cells that have the ability to self-renew, to initiate tumors when transplanted into SCID mice, and to differentiate into nontumorigenic cells, which form the bulk tumor mass [8–11].

The epithelial-mesenchymal transition (EMT) program, normally activated in the early stages of embryonic development, has also been found to play a key role in the early process of metastasis of cancer cells in solid tumors [12, 13]. During EMT, polarized epithelial cells undergo morphogenetic changes and gain the migratory properties of mesenchymal cells [14]. On the molecular level, EMT is governed by aberrantly expressed transcription factors, among which Twist1 and Bmi1 are known to be mutually essential in promoting EMT [15]. Activation of EMT program in cells results in decreased expression of epithelial markers, namely, E-cadherin and EpCAM, and increased expression of mesenchymal markers, namely, N-cadherin and Vimentin [14]. In addition to the fact that EMT allows for enhanced cell motility and invasion, required for tumor progression, a breakthrough in breast cancer first demonstrated EMT can generate tumor cells with stem-like properties [16].

According to the “seed and soil” theory of metastasis development, tumor cells may enter the blood circulation after detaching from the primary tumor and circulate to reach distant organs, where they reattach and give rise to metastases [17]. Lending support to this theory, the presence of circulating tumor cells (CTCs) in blood samples of patients with lung cancer has strong impact on overall survival and can even predict disease recurrence [18–20]. It is speculated that reseeding of malignant cells and consequent metastases can only develop from a restricted population of CTCs, which has undergone EMT and acquired self-renewing capacities coupled with high migratory potential, giving them the ability to migrate to distant sites via blood stream, reimplant and initiate* de novo* tumor growth [7].

The association between EMT and stem-like phenotype in lung cancer cells was shown in several* in vitro* studies [21–24], but the data on this phenomenon in lung cancer patient samples are limited. The aim of our study was to determine expression of EMT-inducing transcription factors (*BMI1*,* TWIST1*), CSC markers (*CD133*,* ALDH1A1*), and epithelial markers (*EpCAM*) in primary tumors and matching whole blood samples as well as in CTCs of patients of NSCLC and to evaluate possible associations between marker expression within primary tumors, within whole blood and between tumors and blood.

## 2. Methods

### 2.1. Study Population

Tumor tissue and whole blood samples were obtained from 57 consecutive patients with operable NSCLC, treated with radical surgery. In addition, 13 systemic therapy-naïve patients with advanced NSCLC for the isolation of CTCs were included. Patients with a history of other malignancies were not eligible. All patients had pathologically confirmed NSCLC and were diagnosed and treated by standard clinical practice valid at that time at University Clinic Golnik between 2012 and 2013. All patients signed an informed consent and the study was approved by the Slovenian National Committee for Medical Ethics, protocol number 40/04/12.

### 2.2. Tumor Tissue and Blood Collection

Tumor tissue for gene expression analysis was collected from primary tumor immediately after surgical resection and stored in RNAlater (Qiagen, Hilden, Germany) at −20°C until RNA isolation. Tumor tissue sampling was performed by skilled pathologist and histological diagnosis was determined based on microscopic features according to the WHO classification. Whole blood samples (2.5 mL) for gene expression analysis were collected from each patient one day after the surgery in PAXgene blood tubes (PreAnalytiX, Hombrechtikon, Switzerland) and stored at −20°C until RNA isolation. Whole blood samples (30 mL) for CTC isolation were collected from advanced NSCLC patients before the beginning of first-line systemic therapy in heparinized tubes and were immediately processed for CTC isolation. All blood samples were obtained after the first 5 mL of blood was discarded to avoid contamination of the blood sample with skin epithelial cells.

### 2.3. CTC Isolation by Magnetic Bead Separation

Peripheral blood mononuclear cells (PBMNCs) were isolated using a Ficoll-Paque density gradient, washed twice with sterile PBS, and resuspended in 300 *μ*L PBS supplemented with 0.5% BSA (Miltenyi Biotec, Bergisch Gladbach, Germany). PBMCs were incubated with 100 mL of magnetic beads coated with anti-EpCAM (CD326) antibody (Miltenyi Biotec) and 100 mL of FcR blocking reagent (Miltenyi Biotec) for 30 minutes at 4°C. Afterwards, PBMNCs were magnetically separated, utilizing MS separation columns and MiniMACS Separator (Miltenyi Biotec) to positively select EpCAM-positive CTCs from the remaining PBMNC fraction (flow through).

### 2.4. Gene Expression Analysis

Total RNA from tumor tissue and from isolated CTCs was extracted using the miRNeasy Mini Kit (Qiagen), according to the manufacturer's instructions. RNA from whole blood samples was isolated with PAXgene Blood miRNA Kit (PreAnalytiX) and purified using the fully automated QIAcube system (Qiagen) to standardize the RNA isolation procedure. All RNA samples were treated with RNase-free DNase (Qiagen) and reverse transcribed to cDNA using the high-capacity cDNA Reverse Transcription Kit (Applied Biosystems, Foster City, CA, USA).

cDNA was quantified by RT-qPCR (ABI PRISM 7500 Fast Real-Time PCR System) at standard conditions utilizing TaqMan Universal PCR Master Mix II or Taqman Gene Expression Master Mix (both Applied Biosystems) for preamplified cDNA samples. The TaqMan assays EpCAM (CD326) (Hs00901885_m1), BMI1 (Hs00180411_m1), TWIST1 (Hs00361186_m1), PROM1 (CD133) (Hs1009245_m1), and ALDH1A1 (Hs00167445_m1) were used. All measurements were taken in triplicate for each sample and the relative expression was analysed using the ΔΔCt method [25]. With this method, the mRNA amounts of the target gene were normalised to an endogenous control and relatively to a calibrator, using the formula RQ sample = 2^−(ΔCt  sample−ΔCt  calibrator)^. We used glyceraldehyde-3-phosphate dehydrogenase (GAPDH; 4333764F) as an endogenous control (Applied Biosystems). A549 cell line was used as calibrator for normalizing gene expression in primary tumors and whole blood, whereas EpCAM-negative PBMNCs (flow through) were used as calibrator for normalizing gene expression in CTCs. All samples with threshold cycle ≥ 38.0 were considered negative.

### 2.5. Immunohistochemistry

Tissue microarrays (TMAs) were constructed from all 57 primary tumor samples. The tissue area for sampling was selected based on visual alignment with the corresponding haematoxylin-eosin (H&E) stained section. Tissue sections of 6 mm were cut from formalin-fixed paraffin embedded (FFPE) tissue blocks of each tissue specimen in triplicate and cut into 5 *μ*m thick sections. IHC detection of BMI1, CD133, and ALDH1A1 was performed according to the manufacturer's instructions and carried out on an automated platform, the Benchmark XT (Ventana Medical Systems, Tucson, AZ, USA). The primary antibodies consisted of a mouse monoclonal anti-CD133 antibody clone AC133 (Miltenyi Biotec, Bergisch Gladbach, Germany; dilution 1 : 50), a rabbit monoclonal anti-ALDH1A antibody clone EP1933Y (Abcam, Cambridge, UK; dilution 1 : 200), and a rabbit monoclonal anti-BMI1 antibody clone EPR3745(2) (Abcam, dilution 1 : 100). Two investigators (Ana Koren and Izidor Kern) independently evaluated the marker expression utilizing light microscopy. Approximate percentage of positive cells over the total number of tumor cells and score 0−3 was assigned for the immunostaining intensity (0: negative, 1: moderate, 2: strong, and 3: very strong). The average values for percentage of stained area were multiplied by average values for intensity in each tissue to derive a composite expression score (expression score = area × intensity). The percentage and intensity of staining were scored for each core separately and then average of expression scores was calculated for each patient. NSCLCs were dichotomized into high and low expression classes on the basis of ALDH1A1 and BMI1 median expression scores, whereas tumors with any detectable CD133 expression were scored high class. Normal kidney tissue was used as positive control for BMI1 and ALDH1A1 staining, whereas a strongly stained sample served as positive control for CD133 in each batch of staining.

### 2.6. Statistics

The distribution of data was determined using the D'Agostino and Pearson omnibus normality test. Spearman's rank correlation coefficient analysis was performed to determine associations between marker expression levels in tumor and matching whole blood samples. Linear regression analysis was performed to demonstrate correlation between markers in tumor tissue samples. Fisher's exact test was performed on the associations between high and low CD133, ALDH1A1, and BMI1 protein expression scores in TMA samples. Statistical analysis was performed using GraphPad Prism software (version 5, San Diego, CA, USA). After Bonferroni correction for multiple comparisons was applied, *P* < 0.01 was considered statistically significant.

## 3. Results

### 3.1. Patient Characteristics

Patients' clinical characteristics are presented in Table 1. The median age of the 57 operable NSCLC patients was 62 (range 42–79 years), more than half of the patients were males (33/57; 58%), 32 out of 57 (56%) had adenocarcinoma, and 19 out of 57 (33.3%) had squamous cell carcinoma. The majority of patients were current or former smokers (46/57; 81%). All patients were diagnosed with the limited stage disease (29/57, 51% stage I; 14/57, 25% stage II; 14/57, 25% stage III) and were treated with radical surgery. Four out of 57 patients received neoadjuvant platinum-based chemotherapy before surgery. The median age of 13 advanced NSCLC patients was 67 (range 50–82), and 6 out of 13 (46.2%) were males. All 13 (100%) had adenocarcinoma histology and were diagnosed with stage IV disease.

### 3.2. Gene Expression Profiles in Primary Tumors and Whole Blood

**Figure 1:**
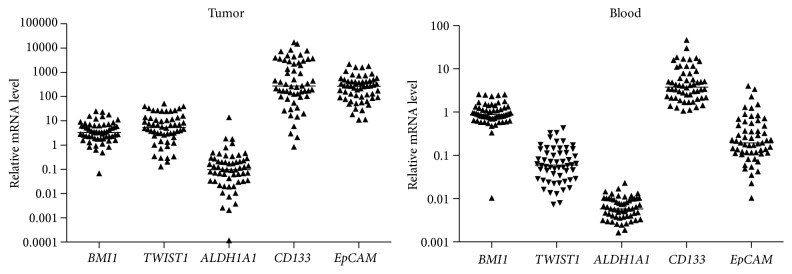
mRNA expression levels of EMT (*BMI1, TWIST1*), CSC (*CD133, ALDH1A1*), and epithelial (*EpCAM*) markers in primary tumors and whole blood samples of 57 NSCLC patients. Data are shown as scattered plot for all measured values with line in the middle representing the median.

Relative gene expression levels of the five studied markers were identified in 56 out of 57 (98%) primary tumors and in 55 out of 57 (96%) whole blood samples.  Figure 1 shows mRNA expression profiles of* BMI1*,* TWIST1*,* ALDH1A1*,* CD133*, and* EpCAM* in tumors and blood. Their relative mRNA expression ranged from 0.0001 to 16797 in tumors and from 0.002 to 46 in blood. Several associations between EMT (*BMI1*,* TWIST1*), CSC (*ALDH1A1*,* CD133*), and epithelial (*EpCAM*) marker expression within tumor, within blood, and between tumor and blood samples were identified (Table 2).

In primary tumors, significant correlations between EMT and CSC markers, namely, between* BMI1* and* ALDH1A1* (*r*_*s*_ = 0.622; *P* = 2 × 10^−7^) and between* BMI1* and* CD133* (*r*_*s*_ = 0.452; *P* = 2 × 10^−4^), were found. No significant correlation between* TWIST1* and* CD133* or* ALDH1A1* (both *P* > 0.01) was observed. In addition, significant correlation between CSC markers* CD133* and* ALDH1A1* (*r*_*s*_ = 0.544; *P* = 1 × 10^−5^) was confirmed. We found no correlation between EMT markers* BMI1* and* TWIST1* (*P* > 0.01). Significant correlations between EMT/CSC and epithelial marker expression were also observed, specifically between* BMI1* and* EpCAM* (*r*_*s*_ = 0.681; *P* = 6 × 10^−9^) and between* ALDH1A1* and* EpCAM* (*r*_*s*_ = 0.475; *P* = 2 × 10^−4^). No correlation between* TWIST1* and* EpCAM* was found (*P* > 0.01).

Relative mRNA expression of studied markers in blood samples was lower, but, still, several associations between studied markers were confirmed. Identically to primary tumors, significant correlations between* BMI1* and* CD133* (*r*_*s*_ = 0.353; *P* = 0.008) and between* BMI1* and* ALDH1A1* (*r*_*s*_ = 0.407; *P* = 0.002) were observed. Again, no correlation between* TWIST1* and* CD133* or* ALDH1A1* (both *P* > 0.01) was found. Contrary to primary tumors, we found no correlation between CSC markers* CD133* in* ALDH1A1* (*P* > 0.01). However, significant correlation between* BMI1* and* TWIST1* (*r*_*s*_ = 0.283; *P* = 0.002) mRNA expression levels was identified in the blood. We also did not confirm any correlation between EMT/CSC markers and* EpCAM* expression (all *P* > 0.01) in the blood.

The investigation for possible associations in EMT/CSC marker expression between tumor tissue and blood samples showed* BMI1* expression in primary tumors positively correlated with* BMI1* expression in whole blood (*r*_*s*_ = 0.35; *P* = 0.008). No other significant correlations in EMT/CSC marker expression between tumor tissue and blood were identified (all *P* > 0.01).

### 3.3. Protein Expressions in Primary Tumors

**Figure 2:**
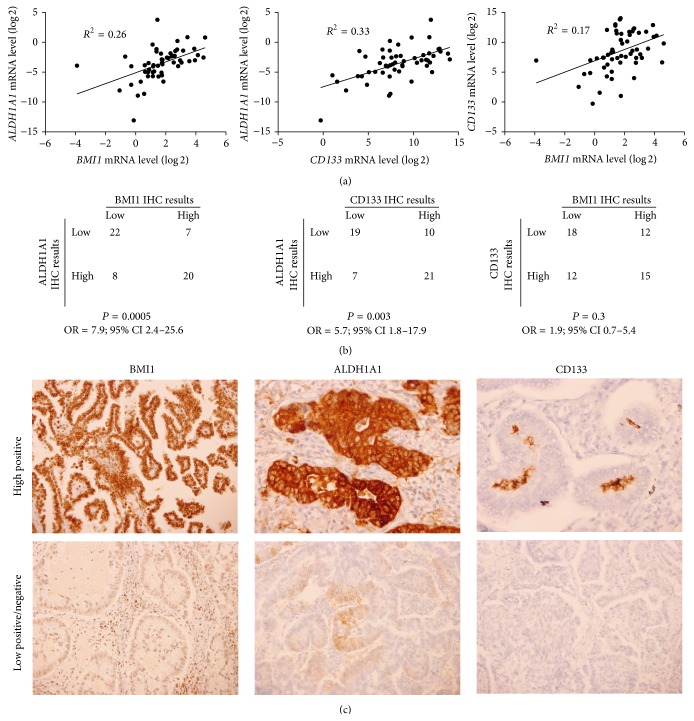
Association between EMT in CSC marker expression in primary-tumors of 57 NSCLC patients. (a) Linear regression results between* BMI1*,* ALDH1A1*, and* CD133* mRNA expression. (b) Correlation of the IHC grading of BMI1, ALDH1A1, and CD133 protein expression. (c) Representative results of IHC stainings for BMI1, ALDH1A1, and CD133 at different staining intensities (top, high positive; bottom, low positive/negative).

To re-test positive association between EMT and CSC markers mRNA expression levels in the primary tumors, measured by RT-qPCR (Figure 2(a)), possible associations between* BMI1*,* ALDH1A1*, and* CD133* protein expression levels in the primary tumor, measured by IHC, were tested. IHC evaluation was performed in all 57 tumor samples. Of these, 28/57 (49%) were classified as positive for BMI1, 27/57 (47%) were classified as positive for CD133, and 28/57 (49%) were classified as positive for ALDH1A1 expression. The BMI1 protein expression was closely associated with ALDH1A1 protein expression (*P* = 5 × 10^−4^). Overall, 20/27 (74%) tumor specimens that showed high BMI1 expression also showed high expression of ALDH1A1. ALDH1A1 and CD133 were also associated (*P* = 0.003); 21/31 (68%) tumor samples that were classified as having high ALDH1A1 expression were also positive for CD133 expression. On the contrary, BMI1 and CD133 protein expression levels in the primary tumors were not shown to be associated significantly (*P* = 0.3), although 15/27 (56%) samples with high BMI1 expression also expressed CD133 (Figure 2(b)). Representative BMI1, ALDH1A1, and CD133 staining results are shown in Figure 2(c).

### 3.4. Gene Expression Analysis in Circulating Tumor Cells

**Figure 3:**
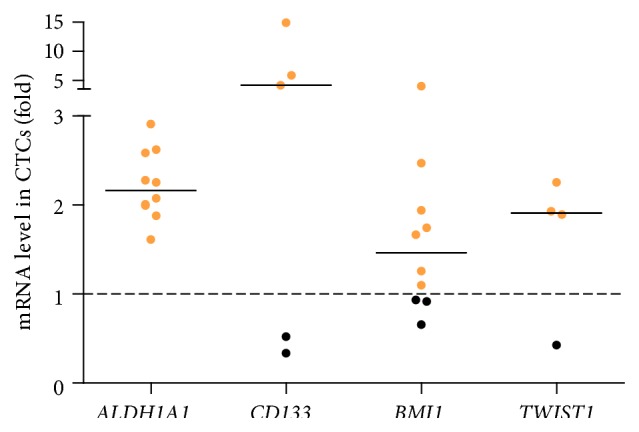
Expression levels of CSC (*CD133, ALDH1A1*) and EMT (*BMI1, TWIST1*) in EpCAM-positive CTCs of 10 patients with metastatic NSCLC. Dashed line represents relative mRNA level in PBMNCs.* ALDH1A1* and* BMI1* transcripts were detected in 10/10 patients whereas* CD133* and* TWIST1* transcripts were detected in 5/10 and 4/10 patients, respectively. Data are shown as scattered plot for all measured values with line in the middle representing the median mRNA expression level of marker in CTCs.

Gene expression of* EpCAM* in CTCs was detected in 10/13 studied patients (median 23.83; range 1.66–63.12), and the molecular profiling for EMT and CSC markers was performed only for this subgroup.* ALDH1A1* relative gene expression in CTCs ranged from 1.61 to 2.91 (median 2.17) and was detectable in 10/10 patients, whereas* CD133* expression in CTCs ranged from 0.34 to 14.94 (median 4.14) and was detectable in 5/10 patients.* BMI1* relative expression in CTCs ranged from 0.66 to 3.99 (median 1.46) and was detectable in 8/10 patients whereas* TWIST1* relative expression in CTCs ranged from 0.43 to 2.25 (median 1.91) and was detected in 4/10 patients. Overall,* ALDH1A1* was overexpressed in CTCs of 10/10 (100%) patients,* CD133* was overexpressed in 3/10 (30%) patients,* BMI1* was overexpressed in CTCs of 7/10 (70%) patients, and* TWIST1* was overexpressed in CTCs of 3/10 (30%) patients (Figure 3).

## 4. Discussion

Evidence shows that tumors are heterogenous, consisting of several cancer cell populations, which contribute differently to tumor maintenance, malignancy, and ability to invade the surrounding environment [26]. The cancer stem cell model provides one explanation for the phenotypic and functional heterogeneity among cancer cells within the given tumor [5]. EMT is considered to be a fundamental mechanism, which promotes invasion and metastasis of tumor cells [14]. Findings presented here demonstrate an association between EMT and CSC marker expression levels in tumor tissue and blood of NSCLC patients. Specifically, we found a significant association between ALDH1A1, CD133, and Bmi1 expression levels, indicating Bmi1 expression is associated with lung CSCs. On the other hand, no significant association between Twist1 and CSC marker expression was found in our study. Our results suggest CSCs are present in both, tumors and blood of NSCLC patients, whereas Bmi1 may play an important role in initiation and maintenance of stemness properties of tumor cells of NSCLC patients and might be involved in blood-borne dissemination of NSCLC tumors.

Lung cancer cellular models suggest EMT can trigger conversion to a CSC phenotype [21–24], but data confirming this phenomenon in actual patients are limited. In the present study, positive correlations between EMT-inducing transcription factor* BMI1* and CSC markers* CD133* and* ALDH1A1* mRNA expression levels were identified in primary tumors as well as in whole blood. This finding suggests that Bmi1 is involved in generation of lung CSCs. Similar observation with regard to Bmi1 enrichment in CD133-positive cells was reported in glioblastoma multiforme primary cells and tumor samples [27]. Analogous studies confirmed Bmi1 expression in CD44-positive head and neck squamous cell carcinoma CSCs [28] and in CD49f Sca-1-double positive prostate CSCs [29]. Bmi1 was proposed to regulate self-renewal of putative lung epithelial stem cells, and was shown to be critical for lung tumor development [30]. In lung cancer, several studies confirmed aberrant expression of BMI1 in NSCLC tumors [31–33], but, to the best of our knowledge, correlation between Bmi1 and CSCs has not been demonstrated until now. In addition, we found a significant correlation between expression of* BMI1* and* EpCAM* in tumors. This result suggests that although EMT seem to occur in tumor tissue, cancer cells preserve epithelial marker expression, which is consistent with the result of Pirozzi et al., who demonstrated coexpression of EpCAM and mesenchymal marker CD90 in approximately 10% of primary tumor cells of NSCLC patients [34].

On the other hand, no significant association between EMT marker* TWIST1* and CSC marker expression was found. This is contrary to the result of Padín-Iruegas et al., who managed to show positive association between* TWIST1* and* CD133* expression in colorectal cancer, yet the study encompassed only 28 patients [35]. Data on Twist1 expression in lung cancer clinical samples are limited; there are no reports on association between Twist1 expression and CSC markers. The result of one study managed to show positive association between Twist1 and another EMT marker N-cadherin in NSCLC tumors [36]. In our study, no significant correlation between* BMI1* and* TWIST1* expression in primary tumors was observed, though cooperation of Bmi1 with Twist1 during EMT has been shown recently [15]. Nevertheless, positive correlation between* BMI1* and* TWIST1* was identified in whole blood, indicating the presence of mesenchymal cells.

Correlation between* ALDH1A1*,* CD133,* and* BMI1* mRNA expression was found also in whole blood, which suggested their expression in blood might be linked to CTCs. To explain this, we isolated EpCAM-positive CTCs from patients with metastatic NSCLC, for whom we assumed to have the highest number of CTCs, enabling us to perform molecular analysis. We found* ALDH1A1* and* BMI1* were expressed in CTCs of majority of patients, whereas CTCs of only few patients expressed* CD133* and* TWIST1*. This result is in accordance with the whole blood analysis results, where we observed significant association between* CD133* and* TWIST1* expression, whereas trends towards positive association between* EpCAM* and* ALDH1A1* (*P* = 0.04) and negative association between* TWIST1* and* EpCAM* (*P* = 0.02) were observed. With our method, which was based on EpCAM expression, we missed the subpopulation of CTCs with mesenchymal characteristics [37], but their presence might be reflected in the whole blood expression analysis. Expression of ALDH1A1 in EpCAM-positive cells could be explained by the dual role of EpCAM as a cell adhesion molecule and also receptor involved in the maintenance of CSC phenotype through the Wnt signaling pathway [38, 39]. The expression of* BMI1* or* TWIST1* in CTCs can be explained by the recently demonstrated ability of breast and NSCLC CTCs to dynamically switch between epithelial and mesenchymal state [40] or even simultaneously express both epithelial and mesenchymal markers [41, 42]. Our results suggest that lung CTCs are enriched for CSCs, which is in line with the result observed in other tumor types, where it was shown that a subpopulation of CTCs in the peripheral blood of liver and breast cancer patients exhibits CSC-associated markers [37, 43] and complies with the recent demonstration that lung CTCs possess tumor initiation capabilities [44].

The development of noninvasive methods to detect and monitor tumors continues to be a challenge in oncology. We did not identify any correlation in CSC marker expression between tumors and blood, but interestingly we found* BMI1* expression in primary tumors to be positively correlated with* BMI1* expression in whole blood. Based on our observations we propose that Bmi1 might function as metastasis initiation gene by promoting EMT and stemness phenotype, which is mirrored in significant correlation between* BMI1* levels detected in tumors and blood. This result has the potential to be clinically relevant, since longitudinal measurements of* BMI1* mRNA levels in whole blood samples of NSCLC patients might reflect the current burden of malignant disease and could potentially serve as an early noninvasive marker of cancer cell spread and evolution through the course of disease.

With the protein expression analysis we were able to confirm BMI1 and ALDH1A1 coexpression and CD133 and ALDH1A1 coexpressions in primary tumors. A possible reason for the lack of association between BMI1 and CD133 protein expressions may lie in low rate of CD133-positive cells within tumors. As already observed by Bertolini et al, CD133-positive cells consist of only 0.02% to 3.5% of all tumor cells, whereas 60% of tumors contain less than 2% of CD133-positive cells [9]. Considering this fact, it might be possible that RT-qPCR is more sensitive method for detecting small numbers of positive cells than IHC.

Possible reason for some inconclusive results in our study might also be a consequence of intratumoral heterogeneity [4]. The samples used for RNA isolation were small and were excised manually from only one tumor site. As CSCs exhibit plasticity and EMT occurs at different proportions in multiple tumor regions, it might be that more tissue sampling sites would give us more accurate result on how EMT and CSC are associated. This issue was partially overcome by the multiple sampling performed in IHC analysis. Also, the molecular analysis of studied markers was performed only in EpCAM-positive CTCs, not allowing us to study CTC subpopulations which express mesenchymal markers only. Lastly, our correlation analysis was performed only between 5 analysed genes; we are aware that a wider panel of EMT and CSC markers would be needed to fully elucidate link between EMT and CSCs in lung cancer.

In conclusion, in the present study we show, for the first time, the significant association between of EMT-inducing transcription factor Bmi1 and CSC marker expression in NSCLC. This association was confirmed not only in primary tumors but also in blood, suggesting the importance of EMT and CSC in blood-borne dissemination of NSCLC. Additional studies are needed to gain mechanistic insight into how Bmi1 is involved in generation of lung CSCs and how its expression levels in whole blood might be utilized as a potential longitudinal biomarker of disease spread and treatment response in NSCLC and wider. Better understanding of how EMT and CSCs govern tumor progression might as well result in development of new treatment approaches, which would potentially target cancer stem cell populations through a selective blockade of the EMT cascade, finally resulting in better survival of NSCLC patients.

## Figures and Tables

**Table 1:** Clinical characteristics of 57 operable and 13 advanced NSCLC patients.

Characteristics	Operable NSCLC *N* (%)	Advanced NSCLC *N* (%)
Age in years: median (range)	62 (42–79)	67 (50–82)
Gender		
Male	33 (57.9%)	6 (46.2%)
Female	24 (42.1%)	7 (53.8%)
Histology		
Adenocarcinoma	32 (56.1%)	13 (100%)
Squamous cell carcinoma	19 (33.3%)	
Others^*∗*^	6 (10.5%)	
Clinical stage		
I	29 (50.8%)	
II	14 (24.6%)	
III	14 (24.6%)	
IV		13 (100%)
Smoking history		
Yes	46 (80.7%)	11 (84.6%)
No	9 (15.8%)	2 (15.4%)
Unknown	2 (3.5%)	

*N*: number of patients; NSCLC: non-small-cell lung cancer. ^*∗*^Three patients had adenosquamous carcinoma, one basaloid carcinoma, one large cell carcinoma, and one combined large cell neuroendocrine carcinoma with adenocarcinoma.

**Table 2:** Spearman's rank correlation coefficient analysis of associations between mRNA expression of EMT (*BMI1*, *TWIST1*), CSC (*CD133*, *ALDH1A1*), and epithelial (*EpCAM*) markers in primary tumors and matched whole blood samples of 57 operable NSCLC patients. Statistically significant associations (*P* < 0.01) between EMT and CSC marker expression are presented in bold.

	Primary tumor					Whole blood				
	*CD133*	*ALDH1A1*	*BMI1*	*TWIST1*	*EpCAM*	*CD133*	*ALDH1A1*	*BMI1*	*TWIST1*	*EpCAM*
Primary tumor										
*CD133*		*r*_*s*_ = **0.544**	*r*_*s*_ ** = 0.452**	*r*_*s*_ = −0.039	*r*_*s*_ ** = **0.32	*r*_*s*_ = 0.052	*r*_*s*_ = 0.079	*r*_*s*_ = 0.097	*r*_*s*_ = 0.067	*r*_*s*_ = 0.159
		(*P* = **1** × **10** ^−5^)	(*P* = **4** × **10** ^−4^)		(*P* = 0.02)					
*ALDH1A1*			*r*_*s*_ ** = 0.622**	*r*_*s*_ = −0.032	*r*_*s*_ ** = 0.475**	*r*_*s*_ = 0.055	*r*_*s*_ = 0.116	*r*_*s*_ = 0.232	*r*_*s*_ = 0.095	*r*_*s*_ = 0.044
			(*P* = **2** × **10** ^−7^)		(*P* = **2** × **10** ^−4^)					
*BMI1*				*r*_*s*_ = 0.014	*r*_*s*_ ** = 0.681**	*r*_*s*_ = 0.220	*r*_*s*_ = 0.186	*r*_*s*_ ** = 0.347**	*r*_*s*_ = 0.119	*r*_*s*_ = 0.119
					(*P* = **6** × **10** ^−9^)			(*P* = **0.008**)		
*TWIST1*					*r*_*s*_ = −0.262	*r*_*s*_ = 0.102	*r*_*s*_ = −0.050	*r*_*s*_ = 0.180	*r*_*s*_ = 0.109	*r*_*s*_ = −0.085
*EpCAM*						*r*_*s*_ = 0.092	*r*_*s*_ ** = **0.293	*r*_*s*_ ** = **0.289	*r*_*s*_ = −0.062	*r*_*s*_ = 0.222
							(*P* = 0.03)	(*P* = 0.03)		

Whole blood										
*CD133*							*r*_*s*_ = 0.225	*r*_*s*_ ** = 0.353**	*r*_*s*_ = 0.283	*r*_*s*_ = 0.181
								(*P* = **0.008**)	(*P* = 0.04)	
*ALDH1A1*								*r*_*s*_ ** = 0.407**	*r*_*s*_ = −0.034	*r*_*s*_ = 0.283
								(*P* = **0.002**)		(*P* = 0.04)
*BMI1*									*r*_*s*_ = **0.406**	*r*_*s*_ = −0.135
									(*P* = **0.002**)	
*TWIST1*										*r*_*s*_ = −0.313
										(*P* = 0.02)
*EpCAM*										

## Abbreviations

PBMNCs: Peripheral blood mononuclear cells
EMT: Epithelial-mesenchymal transition
CSC: Cancer stem cell
CTC: Circulating tumor cell
NSCLC: Non-small-cell lung cancer
ALDH: Aldehyde dehydrogenase
RT-qPCR: Reverse transcription quantitative PCR
TMA: Tissue microarray.
